# FDA-approved medications that impair human spermatogenesis

**DOI:** 10.18632/oncotarget.12956

**Published:** 2016-10-27

**Authors:** Jiayi Ding, Xuejun Shang, Zhanhu Zhang, Hua Jing, Jun Shao, Qianqian Fei, Elizabeth R. Rayburn, Haibo Li

**Affiliations:** ^1^ Department of Reproductive Medicine, Nantong Maternity and Child Health Hospital, Nantong, China; ^2^ Department of Andrology, Jinling Hospital/Nanjing General Hospital Affiliated with Nanjing University School of Medicine, Nanjing, China; ^3^ CLIA Laboratory Director (various laboratories), Birmingham, AL, USA

**Keywords:** spermatogenesis, prescription drugs, FDA labels, dailyMed, drug development

## Abstract

We herein provide an overview of the single-ingredient U.S. Food and Drug Administration (FDA)-approved drugs that affect human spermatogenesis, potentially resulting in a negative impact on male fertility. To provide this information, we performed an in-depth search of DailyMed, the official website for FDA-approved drug labels. Not surprisingly, hormone-based agents were found to be the drugs most likely to affect human spermatogenesis. The next category of drugs most likely to have effects on spermatogenesis was the antineoplastic agents. Interestingly, the DailyMed labels indicated that several anti-inflammatory drugs affect spermatogenesis, which is not supported by the peer-reviewed literature. Overall, there were a total of 65 labels for drugs of various classes that showed that they have the potential to affect human sperm production and maturation. We identified several drugs indicated to be spermatotoxic in the drug labels that were not reported in the peer-reviewed literature. However, the details about the effects of these drugs on human spermatogenesis are largely lacking, the mechanisms are often unknown, and the clinical impact of many of the findings is currently unclear. Therefore, additional work is needed at both the basic research level and during clinical trials and post-marketing surveillance to fill the gaps in the current knowledge. The present findings will be of interest to physicians and pharmacists, researchers, and those involved in drug development and health care policy.

## INTRODUCTION

Reduced, suppressed, or completely lacking spermatogenesis is a common factor associated with male infertility [1]. There are various causes of suppressed spermatogenesis or spermatogenic failure, including genetic defects [2, 3], but acquired factors also play an important role. For example, men who have been exposed to adverse environmental conditions, such as pesticides or radiation, may become infertile. Lifestyle-related factors such as smoking, heavy drinking, and the use of high-temperature saunas, can also contribute to disorders of sperm production [4, 5]. Many therapeutic drugs have also been reported to impair spermatogenesis, leading to temporary or persistent difficulty conceiving. Any drug that does harm to the spermatogonia, Sertoli cells or Leydig cells can influence the processes of spermatogenesis and sperm maturation. Drugs that induce deleterious changes to the microenvironment of the testes or epididymis may also affect spermatogenesis and sperm maturation, leading to adverse effects on fertility.

There have been several reviews published about therapeutic drugs that impair spermatogenesis (for example, see [1, 6–9]). However, all of these previous reviews were based on searches of the peer-reviewed scientific literature. In contrast, in the present report, we used the data available in DailyMed (https://dailymed.nlm.nih.gov/), a federally-maintained database that contains more than 80,000 drug information and warning labels. The database contains current information about > 85% of the FDA-approved drugs, making it the most complete source of data about these agents. Included in these labels is information ranging from the chemical properties and pharmacology (pre-clinical and clinical) of the drugs to the adverse reactions identified in pre-clinical/non-clinical studies, clinical trials and post-marketing surveillance. These labels are required to be updated whenever new findings are made, so they provide the latest data known for the drugs [10]. Importantly, these labels often include information that has not been published. To the best of our knowledge, this is the first comprehensive review of the FDA-approved prescription drugs that affect human spermatogenesis based on the drug labels.

The objective of the present review was to summarize the relevant information about FDA-approved drugs that can impair spermatogenesis based on the data presented in the drug labels in the DailyMed database. A table describing the drugs found to affect human spermatogenesis and their specific impact on spermatogenesis is included (Table 1), which can serve as a reference for medical professionals, health policy makers, and researchers interested in male fertility or drug development. We also briefly discuss the limitations of the current FDA drug labels and reproductive toxicity testing and make recommendations for future studies.

**Table 1:** The FDA-approved drugs that have the potential to impair human spermatogenesis

Drug category*	Generic name of drug	Adverse impact on human spermatogenesis	Supported/refuted by PubMed publications**
Analgesic	Methadone hydrochloride	Decrease in sperm motility and seminal vesicle secretions, abnormal sperm morphology	Supported in humans [12,13]
	Pregabalin	Epididymitis (rare)	Refuted in humans [14]
	Gabapentin	Epididymitis (rare)	Refuted in rats [15], no data for humans
Anti-arrhythmic agent	Amiodarone hydrochloride	Epididymitis (rare)	Supported in rats [16], no data for humans
Anti-bacterial agent	Lomefloxacin hydrochloride	Epididymitis, orchitis (<1% of patients)	No data published for animals or humans
	Nitrofurantoin	Spermatogenic arrest/decreased sperm count (high doses)	Supported in humans [17]
	Dapsone	Orchitis, male infertility	Supported in rats [18], no data in humans
Anticonvulsant	Lamotrigine	Epididymitis (rare)	Supported in humans [19]
Antidepressant	Clomipramine hydrochloride	Epididymitis (infrequent)	Supported in humans [20]
	Levomilnacipran hydrochloride	Epididymitis, seminal vesiculitis (∼4% of males)	No data published for animals or humans
	Paroxetine mesylate/paroxetine hydrochloride	Decreased sperm quality, epididymitis	Supported in humans [21,22]
	Fluvoxamine maleate	Hematospermia	Supported for human sperm in vitro [21]
	Venlafaxine	Orchitis (rare)	No data published for animals or humans
Antihypertensive agent	Nifedipine	Reversible reduction in ability to fertilize ova	Supported in humans [23]
Anti-infective agent	Voriconazole	Epididymitis (<2% of all patients)	No data published for animals or humans
Anti-inflammatory agent	Colchicine	Azoospermia or oligozoospermia	Refuted in humans [24,25], supported in animals [26](stage-specific)
	Cortisone acetate	Changes in the motility and number of spermatozoa	Refuted in humans [27]
	Dexamethasone/dexamethasone sodium phosphate	Changes in the motility and number of spermatozoa	Refuted in humans [28], supported in several animals [29]
	Methylprednisolone/prednisone	Changes in the motility and number of spermatozoa	No specific data published for methylprednisone, prednisone is supported in humans [30]
	Sulfasalazine	Reversible oligozoospermia and infertility	Supported in humans [31]
	Triamcinolone hexacetonide	Changes in the motility and number of spermatozoa	No data published for animals or humans
Antineoplastic agent	Busulfan	Damage to spermatozoa and testicular tissue, azoospermia, testicular atrophy	Supported in humans [32]
	Chlorambucil	Azoospermia (prolonged or permanent)	Supported in humans [33]
	Cyclophosphamide	Interferes with spermatogenesis, testicular atrophy, azoospermia, oligozoospermia	Supported in humans [34]
	Dabrafenib mesylate	Impaired spermatogenesis, decreased sperm count	No data published for animals or humans
	Degarelix	Testicular atrophy	No data published for animals or humans
	Fludarabine phosphate	Damage to spermatozoa and testicular tissue	Supported in humans [35]
	Mercaptopurine	Oligozoospermia	Refuted in mice [36] and humans [37](both used AZA)
	Methotrexate sodium	Oligozoospermia (reversible)	Supported for rodents [38,39], conflicting data for humans ([40](-) vs. [41](+))
	Procarbazine hydrochloride	Azoospermia	Supported in mice [42], unclear for humans (all trials have used combination therapy [43])
	Triptorelin pamoate	Testicular atrophy	Supported in humans [44]
	Vinblastine sulfate	Azoospermia	Supported in rats [45] and *in vitro* for humans [46]
	Vinorelbine tartrate	Damage to spermatozoa	No data published in animals or humans
	Thalidomide	Orchitis	Supported in rabbits [47], no data for humans
Anti-Parkinson agent	Pramipexole dihydrochloride	Epididymitis, orchitis	No data published for animals or humans
Antipsychotic agent	Quetiapine fumarate	Orchitis (infrequent)	No data published for animals or humans
Anti-rejection drug	Everolimus	Azoospermia or oligozoospermia (∼1% of patients)	No data published for animals or humans
Antiviral agent	Delavirdine mesylate	Hematospermia, epididymitis	No data published for animals or humans
	Ganciclovir/ganciclovir sodium	Testicular hypotrophy, aspermatogenesis (dose-dependent)	Supported in rats [48], no data in humans
	Valganciclovir	Inhibition of spermatogenesis	No specific data published, but is expected to result in the same effects as ganciclovir
Cardiovascular agent	Bosentan	Decreased sperm count	No data published for animals or humans
Hormones, hormone substitutes and hormone antagonists	Clomiphene citrate	Testicular tumors	Supported in humans [49], but used to treat infertility (improves sperm parameters; [50])
	Danazol	Decreased spermatogenesis, abnormalities in semen volume, viscosity, sperm count, and motility	Supported in humans [51]
	Dutasteride	Decreased sperm count, semen volume, and sperm motility	Supported in humans [52]
	Finasteride	Decreased ejaculate volume and total sperm per ejaculation (reversible)	Supported in humans [52]
	Flutamide	Interference with testosterone, decreased sperm count	Supported in mice [53], supported in combination with other agents in humans [54]
	Histrelin acetate	Testicular atrophy	Supported in rats [55] and rhesus monkeys [56], no data for humans
	Leuprolide acetate	Suppressed testicular steroidogenesis, testicular atrophy	Supported in humans [57], also may be useful for protecting/restoring fertility following toxic insults [58,59]
	Methyltestosterone	Oligozoospermia, suppressed spermatogenesis	Supported in dogs [60] and rats [61], no data for humans
	Nandrolone decanoate	Inhibition of testicular function, testicular atrophy and oligozoospermia, epididymitis	Supported in humans [62]
	Nilutamide	Testicular atrophy	No data published for animals or humans
	Oxandrolone	Suppressed spermatogenesis, inhibition of testicular function, testicular atrophy, oligozoospermia, epididymitis	Supported in rats [63] and in a human case report [64]
	Oxymetholone	Inhibition of testicular function, testicular atrophy, oligospermia, decreased seminal volume, epididymitis	Supported in mice [65] and rats [66], no data for humans
	Testosterone/testosterone cypionate/testosterone enanthate/testosterone undecanoate	Suppressed spermatogenesis/oligozoospermia, testosterone undecanoate may also cause spermatocele formation	Supported in humans [67–69], but dose-dependent and variable results
Immunosuppressant	Sirolimus	Azoospermia (reversible)	Supported in humans [70]
PDE5 inhibitor	Tadalafil	Decreased sperm concentration	Refuted in humans [71,72]
Peripheral nervous system agent	Cevimeline hydrochloride	Epididymitis	No data published for animals or humans
Radioactive compound	Sodium iodide I ^131^	Impairment of testicular function/transient infertility	Supported in humans [73]

*Some drugs can be classified into multiple categories. They have been classified here on the basis of their most common indication/target

**Although the drugs noted to support the DailyMed labels were all found to affect some aspect of human spermatogenesis, the effect was not always the same as that listed in the drug label

## THE DATABASE SEARCH

The search of the DailyMed database was performed as described in a previous study, with some modifications [11]. We included only FDA-approved human single-ingredient prescription drugs. Over-the-counter (OTC) drugs were excluded because the relevant information was generally not available in the label due to limited testing. The drugs with multiple active ingredients were also excluded to simplify the analysis. When the same agent had more than one route of administration or was produced by different companies, or when the information had been updated at different times, the most recently updated record was used for the review.

The labels for the remaining drugs were downloaded directly from DailyMed in the PDF format on April 22, 2015 and were manually examined to collect all information related to spermatogenesis. We performed a verification of the drug labels on February 16, 2016 to identify any new or missed entries that had been added since the previous search. The PDF documents were manually searched independently by two authors using stemmed keywords related to sperm production and maturation, including “sperm*, “semin*”, “testi*”, “epidid*”, “deferen*”, “Leydig”, “Sertoli”, and “interstitial”. These keywords were used to search for a variety of terms related to spermatogenesis, with many of the stemmed keywords associated with numerous terms, i.e. “sperm*” identified labels containing the words hypospermatogenesis, spermatids, oligo(zoo)spermia, spermato-, spermatozoon, spermatozoa, spermatogonial, post-spermatogonial, spermatoc(o)ele, spermatogenesis, spermaduct, dysspermatism, a(zoo)spermia, aspermatism, and spermatogenic. When there were differences in the descriptions found between the two authors, they discussed the findings with another author, and the group's consensus regarding the findings was used for the manuscript.

## INCLUSION AND EXCLUSION CRITERIA

The criteria for drug inclusion and exclusion were discussed among the authors, and a consensus was reached by all of the researchers in the group. All information related to spermatogenesis, including sperm production in the testicles and sperm maturation in the epididymis, was included. Some symptoms involving pathological changes of the male productive organs, such as Leydig cell tumors, testitis, epididymitis, and orchitis, which are likely to affect sperm production and maturation, were also included in this study.

Some descriptions were excluded from further analysis for the following reasons: (1) Many labels had information relevant to sperm ejection, such as ejaculation disorders, delayed ejaculation, retrograde ejaculation, erectile dysfunction, prospermia, etc., which were not included in this study. We excluded these findings because they are not directly related to sperm production or maturation. Miltefosine, omeprazole and iloperidone are in this category. (2) Some drugs affect the fertility of male offspring of exposed individuals, such as morphine sulfate. These were also excluded from this study. (3) Some records about the effects of sex hormones were excluded unless a change in the sperm count or quality was confirmed, because the focus of this review was spermatogenesis. In addition, while the fact that hormones can have a negative impact on fertility is generally well-recognized, and some drugs (such as those used to treat prostate cancer) are intended to be anti-androgenic, it is still worthwhile to warn patients of the effects on fertility, because they may not know or understand the risk unless they are given explicit information. Including information in the drug warning label, and having physicians and/or pharmacists explain the information to the patients, will be helpful in ensuring that they fully understand their treatment. We have therefore included some hormone-based drugs where it might seem blatantly obvious that they would have a negative impact simply to insure that they were included in the list, which might eventually be used by non-experts.

## DRUGS EVALUATED IN THIS STUDY

As of April 22, 2015, 65,536 drug labels had been deposited in DailyMed. After excluding the labels for over-the-counter (OTC) drugs; drugs used only in animals; drugs that were not yet FDA-approved; and the labels for medicinal foods, medical devices, dietary supplements, cosmetics, bulk ingredients, vaccines, plasma derivatives, diagnostic kits, and similar devices or reagents, a total of 22,724 human prescription drug labels were retained for the subsequent data extraction.

There were multiple labels from different manufacturers for some drugs, as well as drugs intended for different routes of administration, duration of effect, and in different dosage forms. All of the duplicate labels for the same drugs were deleted manually, and only the most recent label was kept for the study. The final list of human single-ingredient prescription drugs included 1,368 drug labels, and these were all examined for information about their impact on sperm production and maturation.

## THE DRUGS MOST LIKELY TO AFFECT HUMAN SPERM PRODUCTION AND MATURATION

A summary of the drugs with labels indicating that they impair human spermatogenesis is provided in Table 1, with the drugs grouped by their most common indication. As shown in the table, human spermatogenesis was reported to be affected by 65 of the 1,368 drugs examined. Hormones, antineoplastic agents and anti-inflammatory drugs were the drugs most often reported to affect sperm production and maturation based on the drug information and warning labels. However, various classes of drugs, such as antibacterial, antiviral and analgesic agents, were also found to affect human spermatogenesis. All of the drugs shown in the table were reported to result in a decrease in the sperm count/concentration, and/or a decrease in sperm motility, or to otherwise have the potential to lead to oligozoospermia, astheno(terato)zoospermia or azoospermia in their official labels.

### The impact of hormones, hormone substitutes and hormone antagonists on spermatogenesis

Hormones, hormone substitutes and hormone antagonists are well-known for their negative impact on male fertility. In addition to changes in libido, these agents also represent a special class of spermatotoxic agents. Spermatogenesis, which is associated with continuous cell proliferation and differentiation, is regulated by reproductive hormones. Follicle-stimulating hormone (FSH) and testosterone (T) secretion are essential for the successful completion of spermatogenesis. Exposure to exogenous testosterone is considered to inhibit spermatogenesis by suppressing signaling *via* the hypothalamic-pituitary-gonadal hormonal axis [1]. Hormones can also affect germ cells by modulating the Sertoli cell function, because these cells express receptors for both FSH and T [74, 75]. All drugs containing male hormones or their derivatives have the potential to impair sperm proliferation and/or maturation, suppress spermatogenesis, and inhibit the testicular function, potentially leading to oligozoospermia. There is extensive evidence for the negative clinical impact of hormones on the fertility of healthy men (see [49, 51, 52, 54, 57, 62, 64, 67–69] for examples), supporting the warnings included in the drug labels. Therefore, patients being prescribed any type of male hormone or derivative should be counseled that these agents may decrease their fertility.

### The spermatotoxicity of antineoplastic agents

Antineoplastic agents are also well-known for their negative impact on fertility [6, 76, 77]. Antineoplastic agents have the potential to damage both germ cells and the supporting Sertoli cells in men, leading to severe oligozoospermia or azoospermia immediately following most courses of chemotherapy. The rapidly dividing differentiating spermatogonia are generally more sensitive to killing by these drugs than are the later-stage, more slowly proliferating cells comprising most of the human body. Most alkylating antineoplastic agents are toxic to stem cells and produce prolonged azoospermia due to their ability to cross-link DNA [6]. Busulfan is considered to be one of the most highly sterilizing antineoplastic agents, because it kills spermatogonial stem cells [78]. In contrast, other antineoplastic agents do not cause azoospermia, or cause only temporary and/or modest reductions in the sperm counts because they kill only the differentiating spermatogonia.

### The effects of anti-inflammatory drugs

Anti-inflammatory drugs are used to treat conditions ranging from acute (contact dermatitis) to chronic (lupus erythematosus) disorders involving the immune system. These agents have a broad variety of targets, ranging from immune cells to cell surface receptors, signaling molecules and receptors. The labels for eight anti-inflammatory drugs indicated that they had the potential to negatively impact spermatogenesis and reported changes in the motility and/or number of sperm. However, not much has been published in the scientific literature regarding the spermatotoxicity of anti-inflammatory agents. In fact, the studies that have been published seem to indicate that the impact of anti-inflammatory agents on fertility may be minimal [79], non-existent [27], or even positive under certain conditions [80]. Of interest, several clinical trials have used anti-inflammatory drugs in an effort to improve the fertility of men with anti-sperm antibodies [81–83]. While these agents (especially methylprednisolone and prednisolone) consistently reduced the production of anti-sperm antibodies, they did not always lead to improvements in the sperm counts or motility, or in the conception rates. Most of the drug labels in DailyMed indicated that the anti-inflammatory drugs led to changes in the motility and number of sperm. While these parameters are commonly used to assess the impact of exposure on fertility, decreases in the motility and number of sperm may be due to the type and timing of the studies performed rather than to spermatotoxicity, as there are relatively high variations in these parameters [84]. Therefore, the true clinical impact of anti-inflammatory agents is currently unclear and should be examined in greater detail.

### The other drugs that affect human spermatogenesis

A variety of other agents were identified to affect spermatogenesis. In fact, almost half of the FDA-approved drugs reported to affect spermatogenesis do not fit into these three main categories, instead ranging across a variety of intended indications. The most common effect of these drugs was epididymitis, although there were various other causes of spermatogenic failure induced by these drugs, many of which have been substantiated in peer-reviewed publications. For example, tricyclic antidepressants and selective serotonin reuptake inhibitors (SSRIs), such as clomipramine and paroxetine, can lead to significant but reversible suppression of spermatogenesis [20–22]. Some drugs have the potential to induce substantial elevation of the serum prolactin level, which suppresses the gonadotropin-releasing hormone (GnRH) and luteinizing hormone (LH) secretion. Most antipsychotic agents block dopamine in the CNS, leading to suppression of the hypothalamic-pituitary-gonadal axis, thereby affecting hormone signaling and subsequent spermatogenesis [85]. Although detailed mechanistic data is still lacking for most drugs, there does not appear to be any relationship (structural, mechanism of action, target(s), other side effects, etc.) among most of these drugs, reflecting the sensitivity of human reproduction to various insults.

## AGREEMENT OF THE PEER-REVIEWED LITERATURE WITH THE INFORMATION INCLUDED IN THE DAILYMED DRUG LABELS

As noted above, the information reported in the DailyMed drug labels is not always consistent with the published literature. These differences may be due to a lack of publication in peer-reviewed journals, publication bias, differences in the populations studied, differences in the endpoints used for the studies, differences in the dose or frequency of drug administration, or various other factors. We performed a search of the PubMed database (https://www.ncbi.nlm.nih.gov/pubmed/; April 20, 2016) to determine how common such disparities were (see the last column of Table 1). Of the 65 drugs identified to have effects on human spermatogenesis based on the DailyMed labels, there were 13 without any peer-reviewed information available about their effects on spermatogenesis in either animals or humans. Another seven drugs only reported data for animal studies, with no published information available about the effects of the drugs on human spermatogenesis. Therefore, nearly one-third of the drugs indicated to have spermatotoxic effects in humans in the DailyMed drug labels would not be found by a search of the peer-reviewed literature.

With regard to differences in the conclusions of the studies, the peer-reviewed reports for seven drugs were found to contradict the information present in the DailyMed labels. Four of the drugs had contradictory findings in human studies (pregabalin, colchicine, cortisone, and dexamethasone [14, 24, 25, 27, 28]), and one drug had contradictory findings in a study performed in rats (gabapentin [15]). The pro-drug for another agent (AZA, which is metabolized to 6-mercaptopurine) was reported to have no effects in humans or animals [36, 37], and there was conflicting data in the literature regarding the effects of methotrexate in humans [40, 41]. The details of the studies that provided the data for the DailyMed labels are not available, so it is unclear whether there were differences in the study design and methods that might have been responsible for the different findings.

## REPRODUCTIVE TOXICITY TESTING, THE CLINICAL IMPACT OF SPERMATOTOXICITY, AND THE LIMITATIONS OF THE CURRENT DRUG TESTING SYSTEM

### Reproductive toxicity testing

The U.S. FDA requires reproductive toxicity testing of almost all new drugs during development. In the guidelines for industry issued by the FDA for new drug development, it is suggested that reproductive or developmental toxicity be evaluated in a variety of relevant studies to estimate the likelihood of risk to humans. The suggested endpoints include any damage to the reproductive organs; alterations in the endocrine regulation of gamete maturation and release; changes in the sperm count, motility or morphology; alterations in endocrine function; or an overall reduction in fertility [86]. Findings suggestive of these conditions should therefore appear in the drug labels to guide the selection of treatment for patients.

For adverse reactions observed in clinical trials and post-marketing surveillance, including effects on spermatogenesis, the FDA guidelines for industry indicate that the side effects should be presented in the labels under the ADVERSE REACTIONS section [87]. The FDA thus requires the testing and reporting of the impact of drugs on fertility. Most agents are tested at the pre-clinical, clinical, and post-marketing levels, and significant results are subsequently reported in the drug labels.

Despite these guidelines, many of the drug labels currently lack specific information regarding the impact of the drug on spermatogenesis. For example, although approval for a new drug generally requires pre-clinical/non-clinical (animal or *in vitro*) testing, not all FDA-approved drugs have been tested. In some cases, this is because there are no adequate models to test the effects of that drug. In other cases, the drug has been used historically and is generally recognized as safe, permitting its use without stringent testing. In other cases, there may be animal data, but insufficient human data due to small numbers of male patients being treated with the drug, an inability to test for spermatotoxicity (for example, the treatment may only be administered to patients whose sperm production is already comprised due to disease or other treatments), or due to other factors, including the difficulty in obtaining semen samples from patients, the general toxicity of some drugs that makes it impossible to interpret their specific impact on spermatogenesis, or the fact that the timing of patient follow-up visits sometimes misses effects on sperm.

### The clinical impact of spermatotoxicity

Infertility affects approximately 10-15% of the population in developed countries, with about half of the cases identified as being caused by male factor infertility related to abnormal semen production [88]. The decreased fertility in these men may be caused by genetic differences, drug or alcohol abuse, dietary issues, environmental or occupational exposure to toxicants, therapeutic drugs and various other causes. Due to the large number of possible factors underlying male factor infertility, it is currently impossible to estimate the number of men whose fertility is clinically affected by therapeutic agents. However, during the period from 2009-2012, approximately half of the U.S. population (48.7%) had taken a prescription drug in the last 30 days, with more than 20% of the population (21.8%) having taken three or more prescription drugs during the past 30 days [89]. This indicates that a substantial number of men are at risk of experiencing a decrease in fertility due to the spermatotoxic effects of prescription drugs.

### The limitations of the current testing and reporting of adverse effects on spermatogenesis

As noted above, the current reproductive toxicity testing required for drug approval may not identify all drugs with spermatotoxic effects. Rodents are the animals most commonly used for toxicity testing, including for adverse effects on the reproductive system. Although the findings in one mammalian species can usually be generalized to other mammals, there are some differences between species that make it difficult to interpret the findings. For example, mice and rats have a shorter turnover of the epithelial cells lining the seminiferous tubules than do humans, and the total period of time required for spermatogenesis is almost twice as long in humans as it is in mice and rats [90]. This means that the epithelial cells and spermatozoa are exposed to the drug for much longer in men than in rodents. In addition, while testosterone appears to be sufficient to maintain rodent spermatogenesis, both T and FSH are required during the process in humans [90], suggesting that human spermatogenesis may be more sensitive to hormonal perturbations. Although extrapolation factors have been determined to take into account many of the inter-species differences to allow for a prediction of the effects in man, the effects of drugs on spermatogenesis in animals may be different from those in humans, and may therefore yield different findings.

Beyond the species-related differences, another possible explanation for a lack of positive findings in humans is the high variability in spermatogenesis. Even healthy men not taking any medications exhibit daily, monthly and seasonal variations in sperm counts and motility, and the sperm parameters are highly dependent on the length of abstinence prior to ejaculation [84, 90]. In fact, it has been reported that the sperm concentration may vary by as much as 39% in individuals [90]. More detailed and frequent monitoring of the changes in sperm (concentration, number, motility, morphology, DNA integrity, etc.) may help to identify additional drugs that affect male fertility that might not have been detected in studies that examined only a single time point or limited time points after starting treatment. Better study protocols should be designed and added to the guidelines for testing so that adequate information can be collected.

Another important limitation of the present system is the reporting of the results. Although DailyMed is free to use and a relatively user-friendly way to search for the adverse effects of prescription drugs, the majority of the drug labels lack detailed information about the impact of the drugs on spermatogenesis. A few drug labels list the percentages of affected patients, but most list only the type of adverse effect(s) observed (epididymitis, oligospermia, etc.). There is no information included about the number of patients evaluated, the number affected, the population studied or any of the myriad conditions that might have affected the results. Disclosure of the precise findings and details about the treatment and study protocols would be useful. A “level of evidence” system could be implemented that would take into account the total number of patients evaluated, the type of study (whether there were controls, randomization, how often the endpoints were evaluated, etc.), and whether the results were confirmed in an additional study in humans or in animal studies. Such a system would be useful to aid in decision-making regarding the risk of spermatotoxicity.

Some of the drugs with positive information indicating that they affect spermatogenesis indicate in their labels that physicians should provide advice to the patients who will receive treatment. For example, the drug label stipulates that when a patient is going to receive busulfan, physicians should recommend that he use effective contraception during and after the treatment, because the drug damages spermatozoa, resulting in possible genetic abnormalities in a fetus. Similar recommendations should be clearly made for all drugs with potential adverse effects.

The ability to regain normal spermatogenesis following spermatotoxic treatment varies depending on the agent used, the dose, the length of (or number of cycles of) treatment and various other factors. The sperm count and potential fertility are expected to fully recover in many patients within three months after the cessation of drug treatments that temporarily affect sperm production [8, 78]. However, few drug labels currently provide an expected time course for recovery, and this endpoint is not generally included in either animal or human reproductive toxicity testing. This makes it difficult for physicians and pharmacists to provide advice regarding whether the patient receiving treatment can expect to regain full potency, and when/if normal fertility can be expected after the cessation of treatment. Further research is needed to determine the recovery period required before normal fertilization can be expected (if ever) for all drugs with a negative impact on fertility. In addition, new regulations should be implemented regarding the inclusion of long-term endpoints to assess recovery as part of clinical trials. In addition, new recommendations should be made regarding the advice or educational materials that should be given to patients taking drugs with the potential to affect human fertility.

## RECOMMENDATIONS TO IMPROVE THE TESTING FOR SPERMATOTOXICITY

There have been numerous proposals describing ways to improve the reproductive toxicity testing of new agents [90–93]. Some of these have already led to changes, but the majority of toxicology testing has remained the same for decades. The most important steps that should be taken to better assess the risk of spermatotoxicity are: 1) the use of more appropriate animal and/or *in vitro* models, 2) the incorporation of more frequent and repeated sampling to assess the impact of chronic treatment, 3) the development and implementation of better mathematical and statistical methods to analyze the results and predict the human clinical impact of treatment, 4) improved protocols for human clinical trials that allow for the adequate assessment of parameters related to spermatogenesis, and 5) long-term follow-up to assess the full impact of treatment, including the recovery period for spermatogenesis (when applicable).

## LIMITATIONS OF THIS REVIEW AND FUTURE DIRECTIONS

There are several limitations associated with this review. Some of these are imposed by the drug labels themselves, because it is likely that some drugs with spermatotoxic effects were not identified due to insufficient studies or a lack of reporting. Moreover, some of the findings included in the drug labels are difficult to interpret. For example, although the current drug label in DailyMed indicates that pregabalin leads to epididymitis, which can negatively impact sperm production, a study published in early 2015 concluded that pregabalin did not negatively impact sperm production in healthy volunteers [14]. It is therefore unclear whether the percentage of affected patients (reported to be ∼1% in the drug label) was too low to lead to a significant finding in the recent clinical trial, or whether the epididymitis in these patients was not associated with decreased spermatogenesis. There were other contradictory findings in the literature (as described above) compared to the labels that need to be clarified.

Another limitation of this review is that we examined only single-agent, FDA-approved drugs. There are numerous combination therapies, supplements, complementary and alternative medicines (CAM), and OTC drugs that are regularly taken by patients. Information about the potential reproductive toxicity of these agents is largely lacking, despite the fact that their impact may be clinically relevant. Moreover, many patients take supplements, CAM, and OTC drugs concurrently with prescription drugs (and often fail to inform their physician about their use), potentially complicating the interpretation of toxicology studies.

In addition, this review only profiled the drugs that can affect spermatogenesis. This is a limitation of the study, because drug-induced male infertility can affect a wide variety of processes and steps, from spermatogenesis to the successful birth of a term fetus. The information provided here only refers to the first step. The psychological factors affecting male fertility, which may be altered by treatment with various agents, were also excluded. Therefore, much more information is needed to fully understand the reproductive implications of the drugs currently approved for human clinical use.

More efforts are needed to expand the knowledge about how various drugs affect male fertility. This may include the development of more relevant animal, *in vitro* and/or *in silico* models to pre-clinically assess or predict the potential impact on fertility, as well as more rigorous regulatory requirements for additional types of testing, and more detailed and repetitive testing for reproductive toxicity in both animals and human clinical trials to ensure that all effects are observed. More rigorous monitoring of human spermatotoxicity during clinical trials and post-marketing surveillance is also needed, particularly long-term assessments of the impact on sperm. As more is learned about the entire process of spermatogenesis and fertilization, and the various factors that determine male fertility, additional endpoints can be added to both more accurately define any adverse effects and to determine the precise mechanism of action by which a drug exerts these effects. Such information may make it possible to prevent or overcome adverse effects on spermatogenesis, allowing patients to be treated without a negative impact on their fertility.

To the best of our knowledge, this is the first review to provide information regarding the drugs that impair human spermatogenesis based on a comprehensive search of the FDA-approved single-ingredient drug labels. We reviewed the drugs with reported effects on sperm production and maturation based on the findings of reported clinical studies and post-marketing surveillance (including case reports) that were deposited in the federal database for drug labels (DailyMed). This information may be useful for the pharmaceutical industry during the drug development process, because it may inform future studies of adverse effects, or may provide a better understanding of the potential types of toxicity for specific classes of agents. It will also be useful for governmental regulatory agencies to revise the industry guidelines.

In addition, the present review provides a comprehensive list of the FDA-approved drugs that have been documented to affect spermatogenesis or to cause issues that are frequently associated with decreased sperm quality or quantity. This is particularly useful for basic researchers, because it may suggest new avenues of research to determine the mechanisms of action and to potentially develop strategies to prevent or decrease the reproductive toxicity of these agents (or to develop new contraceptive agents). It may also serve as a reference for clinicians, providing better information for them when they counsel patients prior to treatment with drugs that have a definite or potential risk of impairing spermatogenesis. Finally, this review provides an illustration of the current limitations of drug testing, ranging from the types of tests performed to the way the data is reported, which may help to bolster efforts to improve the system.
